# The Health Impact of Nighttime Eating: Old and New Perspectives

**DOI:** 10.3390/nu7042648

**Published:** 2015-04-09

**Authors:** Amber W. Kinsey, Michael J. Ormsbee

**Affiliations:** 1 Institute of Sports Sciences & Medicine, Department of Nutrition, Food and Exercise Sciences, Florida State University, Tallahassee, FL 32306, USA; E-Mail: awk10d@my.fsu.edu; 2 Discipline of Biokinetics, Exercise and Leisure Sciences, University of KwaZulu-Natal, Durban 4041, South Africa

**Keywords:** nighttime eating, health, metabolism

## Abstract

Nighttime eating, particularly before bed, has received considerable attention. Limiting and/or avoiding food before nighttime sleep has been proposed as both a weight loss strategy and approach to improve health and body composition. Indeed, negative outcomes have been demonstrated in response to large mixed meals in populations that consume a majority of their daily food intake during the night. However, data is beginning to mount to suggest that negative outcomes may not be consistent when the food choice is small, nutrient-dense, low energy foods and/or single macronutrients rather than large mixed-meals. From this perspective, it appears that a bedtime supply of nutrients can promote positive physiological changes in healthy populations. In addition, when nighttime feeding is combined with exercise training, any adverse effects appear to be eliminated in obese populations. Lastly, in Type I diabetics and those with glycogen storage disease, eating before bed is essential for survival. Nevertheless, nighttime consumption of small (~150 kcals) single nutrients or mixed-meals does not appear to be harmful and may be beneficial for muscle protein synthesis and cardiometabolic health. Future research is warranted to elucidate potential applications of nighttime feeding alone and in combination with exercise in various populations of health and disease.

## 1. Introduction

Nighttime eating, particularly before bed, is a topic that has received considerable media attention in recent years. Over the past decades it was thought that health and weight conscious individuals should limit and/or avoid food in the hours close to nighttime sleep because it would negatively impact health and body composition. Ultimately, this may increase the risks for cardiometabolic diseases such as obesity and diabetes. However, recent studies investigating the impact of pre-sleep nutrient intake have reported positive physiological outcomes in various populations. This review will examine the factors that have contributed to previous opinions on the health impact of nighttime eating and explore new evidence suggesting that our understanding of nighttime eating may need to be modified based on the content and quantity of the food consumed during this time.

## 2. Effect of Nighttime Eating: An Old Perspective

The arguments in favor of limiting and/or avoiding food intake late in the night are supported by data demonstrating diurnal variations in glucose tolerance [1], gastric emptying [2], resting energy expenditure [3]. Moreover, in healthy normal weight men, it has been demonstrated that the postprandial metabolic response to identical meals changes throughout the day [4,5]. For instance, when identical meals (~544 kcals; 15% protein, 35% fat, 50% carbohydrate) are consumed either in the morning, afternoon, or night, the thermic response to that meal appears to be the lowest with nighttime intake [4]. Similarly, studies in free-living healthy adults have shown that meal satiety also varies with time of day and that food intake during the night is less satiating and leads to greater daily caloric intake compared to food consumed in the morning hours [6,7]. Collectively, these studies demonstrate that the fate of ingested nutrients changes throughout the day and that nighttime intake, when compared to daytime intake, may lead to over-eating and weight gain with potential metabolic consequences. In light of this, it has been suggested that food consumed at night, prior to sleep, may have adverse effects on health.

While data from animal studies appear to support this concept [8,9], not all studies concur [10]. In human trials, populations of shift workers [11], those with the Night Eating Syndrome (NES; consume a large percentage of total daily calories after dinner) [12], and epidemiological data [13,14,15,16,17] suggest that consuming a majority of daily nutrients in the evening may have adverse health consequences (see the following references for thorough reviews on shiftwork [11,18] and NES [19])). Briefly, regarding shiftwork (characterized by irregular work hours to provide service throughout a 24 h day), available data suggest that shiftwork, but more so night shiftwork, is a risk factor for negative health outcomes [11,18]. For example, it has been shown that night shift workers tend to have a higher prevalence of overweightness, abdominal obesity [20], elevated triglycerides, dyslipidemia, impaired glucose tolerance [21,22,23,24,25], and decreased kidney function [26] compared to day workers. In a study of simulated night shiftwork in normal weight women, reductions in both total daily energy expenditure and the thermic effect of dinner were highlighted as contributing mechanisms for weight gain, obesity and impaired health as a result of shiftwork [27]. Thus, these data demonstrate an increased cardiometabolic risk with night shiftwork.

Similarly, NES is associated with obesity in some studies [16,28,29]. It is unclear, however, as to whether obesity is a consequence or cause of NES. Compared to those who do not eat late at night, individuals with NES consume a larger portion of their total calories at night (≥50% after dinner) and have higher overall daily and evening caloric intake in some [30,31], but not all cases [32,33,34]. Likewise, those with NES have higher 24 h respiratory quotient indicative of greater carbohydrate oxidation and less fat oxidation [35] compared to those without NES. In addition, those with NES consume double the amount of carbohydrate and protein and four times the fat [32] in daily meals compared to those without NES. It is practical to assume that these behaviors would predispose night eaters to weight gain, particularly if food is consumed in chronic excess [36]. However, not all studies report differences in daily caloric intake between night eaters and controls [32,33,34] and, it is interesting to note that NES is also present in normal-weight/non-obese individuals [12,30,31,37,38]. The possibility of the non-obese night eaters engaging in compensatory weight maintenance behaviors (e.g., exercise) cannot be ignored [31] as this would be a plausible explanation for any weight-related differences observed. It is also possible that NES may be a precursor to obesity as one study found that the only difference in NES characteristics between obese and non-obese adults was that the non-obese night eaters were significantly younger [37] suggesting that in the long-term, NES may lead to obesity. Others have found that night eating impairs weight loss attempts [39] and is only associated with weight gain in already-obese individuals [13].

These data from shift workers and NES populations provide some evidence to suggest that consuming the majority of daily nutrients late in the evening may have health consequences. However, this concept cannot be fully understood without considering, the influence of sleep, or lack thereof. An inverse relationship exists between sleep duration and body mass index with a greater likelihood of obesity in those reporting less than 7–9 h of sleep each night [40]. Indeed, both shift workers [41] and individuals with NES [30] report higher levels of subjective sleep disturbances (short sleep, reduced sleep quality, difficulty falling asleep) compared to their respective counterparts. Sleep duration plays a significant role in human behavior [42] and, while speculative, when the duration of sleep is short it is likely that there are just more opportunities (awake hours) to eat and, in the long term, promote unfavorable body composition changes.

Indeed, some epidemiological data suggests that consuming a higher proportion of calories later in the day, as opposed to earlier in the day, is associated weight gain [13,14,15,16,17]. However, not all studies agree [12,36,38,43]. It is important to note that several inconsistencies exist in the research examining the effect of late evening caloric intake and body weight. Some of these discrepancies include, but are not limited to: (1) differences in the calories consumed within a specified time frame (*i.e.*, intake after 5 pm [44] *vs.* 8 pm [14], (2) whether it constitutes a portion of the dinner meal [12] or solely post-dinner intake, and (3) whether the individual wakes up from sleep to eat [12]. Despite these inconsistencies it is evident that consuming large quantities of food (binge eating) in the late evening may have adverse health implications.

## 3. Effect of Nighttime Eating: A New Perspective

As demonstrated above, consuming large meals or the majority of daily nutrients late in the evening may increase susceptibility to obesity and other cardiometabolic diseases. While this may hold true when large quantities of food intake occurs at night, data is beginning to mount to suggest that this finding is not consistent if the food choice is altered to favor small, nutrient-dense, low energy foods and/or single macronutrients (<200 kcals) [45,46,47,48,49,50]. In fact, recent studies have examined the impact of low-energy nutrient intake that occurs in close proximity to sleep and reported positive findings [45,46,47,48,49,50]. Conversely, one study [51] compared the impact of a 200 calorie snack (carbohydrate, 20.6 ± 2.6 g; protein, 2.6 ± 1.1 g; fat 11.0 ± 1.0 g) consumed in the daytime (1000 h) or nighttime (2300 h) for 13 days in healthy, normal weight women (*n* = 11, age, 23 ± 1 years, BMI 20.6 ± 2.6 kg/m^2^). It was reported that despite no difference in nutrient composition or calorie intake for each 13 day period, the nighttime eating resulted in small decreases in 24 h fat oxidation and small increases in total cholesterol [51]. The short duration of this study does not allow for conclusions to be drawn with regard to body composition changes, however, body weight was unchanged. Nevertheless, as there is typically a long duration between eating dinner, sleep, and the next main meal (*i.e.*, breakfast the following morning), the overnight period may represent a 6–8 h window of opportunity to potentially optimize health, metabolism and overall human performance. Fortunately, short-term preliminary studies have provided insight to the potential benefits of pre-sleep nutrient intake and its relevance to healthy, active young [47,52] and older individuals [45] and diseased populations [46,48,49,50,53,54] (Table 1).

### 3.1. Young, Active Individuals

It is well known that acute improvements in muscle protein synthesis, glycogen resynthesis, anabolic hormones, and performance outcomes are optimized when nutrients are consumed in close proximity to exercise (*i.e.*, before, during, or immediately after exercise) [55,56,57,58] rather than delaying intake for hours before and after exercise [59,60]. Of interest, but lacking scientific support, is whether the consumption of nutrients prior to sleep has the potential to augment physiological adaptations and outcomes, perhaps when combined with traditional nutrient timing strategies. The benefits of a nocturnal supply of nutrients during the overnight period may have a pivotal role in sport and performance nutrition as active individuals, athletes, and fitness enthusiasts alike are constantly looking for ways to maximize physiological adaptations, achieve optimal body composition, and improve performance.

**Figure 1:**
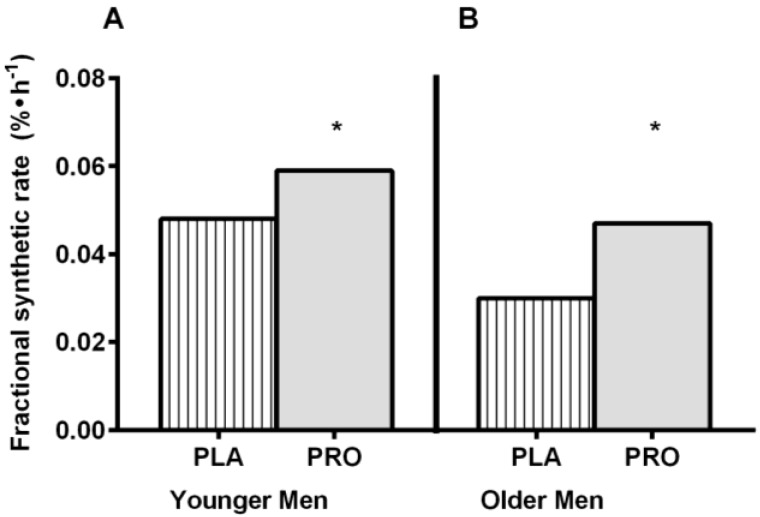
Overnight Mixed Muscle Protein Synthesis in Younger (**A**) and Older Men (**B**). PLA, placebo; PRO, protein. This figure was adapted and redrawn from Res *et al.* [52] and Groen *et al.* [45]. These studies determined mixed muscle protein fractional synthetic rate using l-[ring-^2^H_5_]phenylalanine enrichment as a precursor. * indicates significant difference from PLA.

Protein intake prior to sleep is commonplace among active individuals but until now, evidence-based outcomes from this practice were nonexistent [47,52]. Res *et al.* [52] was the first to investigate whether casein protein consumed before sleep could improve post-exercise recovery. Following a full day of dietary standardization, sixteen recreationally active males performed a single 45 min bout of resistance type exercise in the evening (2000 h). Immediately following exercise all participants were given identical post-exercise beverages (60 g carbohydrate, 20 g whey protein). Approximately 30 min before sleep (~2.5 h post-exercise) participants received either 40 g of casein protein (160 kcals; intrinsically l-[1-13C]phenylalanine-labeled casein obtained from Holstein cow infusion) or a non-caloric placebo beverage. Compared to the placebo group, those receiving the casein before sleep had higher plasma essential amino acid concentrations indicating that protein ingestion before sleep was effectively digested and absorbed. The increase in amino acid availability translated to higher whole-body and muscle protein synthesis rates (~22%, Figure 1A) and a net positive protein balance during the overnight period in the group receiving protein compared to the placebo control [52].

**Table 1:** Effects of small meals/snack consumed at night.

Author	Design	Subjects	Food/Macronutrient	Outcome
Waller *et al.* [46]	Consume cereal with 2/3 cup of fat-free milk at least 90 min post-dinner *vs.* or no-cereal for 4 weeks	Overweight and obese adults	Cereal (100–135 kcals) with low fat milk (~60 kcals)	↓ Body weight (−0.84 ± 1.61 kg)
Groen *et al.* [45]	Single dose of CAS during sleep via nasogastric tube	Elderly men	40g CAS (160 kcals)	↑ Muscle protein synthesis ↓ Hunger (the following morning)
Res *et al.* [52]	Acute resistance exercise bout (2000–2100 h) followed by single dose of CAS 2.5 h post exercise (2330 h) and then sleep (2400 h)	Recreationally active men	40g CAS (160 kcals)	↑ Muscle protein synthesis
Hibi *et al.* [51]	Consume a snack during the day (1000 h) or night (2300 h) for 13 days	Normal weight women	200 kcal snack (20g CHO, 3 g protein, 11g fat)	↓24 h fat oxidation ↑ Total & LDL cholesterol
Madzima *et al.* [47]	Single dose of WH, CAS, CHO or Placebo consumed 30 min before bed	Physically active men	CHO (33 g, 150 kcals) WH (30 g, 150 kcals) CAS (30 g,140 kcals) Placebo (0 kcals)	↑ Morning metabolism with CHO, WH, CAS
Kinsey *et al.* [48]	Single dose of WH, CAS, CHO consumed 30 min before bed	Overweight and obese women	CHO (33 g, 150 kcals) WH (30 g, 150 kcals) CAS (30 g,140 kcals)	↑ Morning insulin in all groups ↓ Hunger (the following morning)
Figueroa *et al.* [50]	Single dose of WH, CAS, CHO consumed 30 min before bed and exercise training for 4 weeks	Obese women	CHO (33 g, 150 kcals) WH (30 g, 150 kcals) CAS (30 g,140 kcals)	↓ Blood pressure ↓ Arterial stiffness
Ormsbee *et al.* [49]	Single dose of WH, CAS, CHO consumed 30 min before bed and exercise training for 4 weeks	Overweight and obese women	CHO (33 g, 150 kcals) WH (30 g, 150 kcals) CAS (30 g,140 kcals)	↑ Morning satiety with CAS Greater magnitude of ↓ body fat and fat mass with WH (non-significant)

Notes: WH, whey protein; CAS, casein protein; CHO, carbohydrate; ↑ , increase; ↓ decrease; all studies were randomized, controlled, trials.

**Figure 2:**
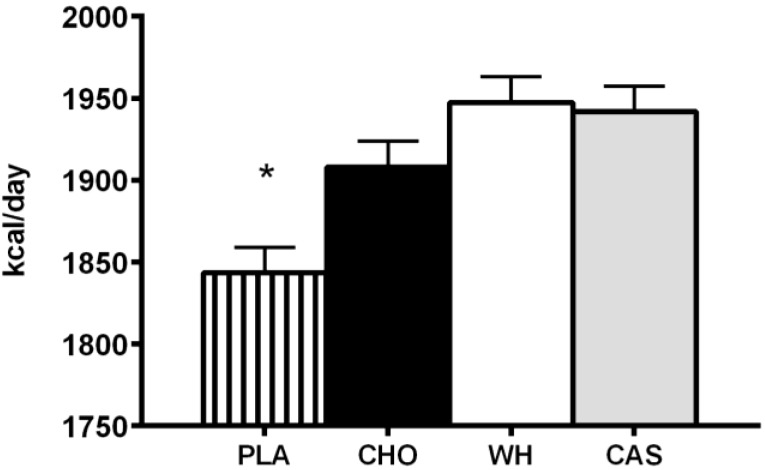
Resting Energy Expenditure Following Nighttime Macronutrient Ingestion in Young Active Men. PLA, placebo; CHO, carbohydrate; WH, whey protein; CAS, casein protein. This figure was adapted and redrawn from Madzima *et al.* [47]. * indicates significant difference from CHO, WH, and CAS.

Casein protein has been suggested to be best before sleep as it is a slow-release protein [61] and may prolong the anabolic milieu, however, the lack of available data comparing the effect of different macronutrients and proteins consumed before sleep limits the ability to draw specific conclusions. Recent work from Madzima *et al.* [47] were the first to compare different macronutrients (carbohydrate *vs.* protein) and proteins (casein *vs.* whey) to a non-caloric placebo consumed prior to sleep on next-morning satiety and metabolism, independent of an exercise stimulus, in healthy, physically active young men. This study had a randomized, double blind, crossover design with treatments separated by 48–72 h. Supplements were provided in beverage form at ingested at least two hours after dinner but within 30 min of going to bed. The authors reported no differences between casein (140 kcal; 30 g micellar casein, 3 g carbohydrate, 0.5 g fat), whey (150 kcal; 30 g whey protein with a 50% blend of whey protein isolate and concentrate, 4 g carbohydrate and 1.5 g fat), or carbohydrate (150 kcal; 0 g protein, 34 g maltodextrin and 2 g fat) in satiety or metabolism. Interestingly, it was reported that consuming a caloric beverage prior to sleep, regardless of type, increased resting energy expenditure, measured the following morning, compared to a non-caloric beverage (Figure 2) [47].

The role of pre-sleep nutrition in active individuals is a largely unexplored area and available data are limited. However, taken together, the data suggest that it may be advantageous for active individuals to consume a small, nutrient dense, high protein beverage (~150 kcals) before bed [47,52]. It is plausible to hypothesize that acute enhancements in overnight muscle protein synthesis and next morning resting metabolism may further aid in the maintenance of and/or improvement in body composition and thereby provide a competitive advantage in healthy, physically active individuals. While this is exciting, data does not exist to support or refute these ideas. However, it is clear that longer-term studies are warranted to see if meaningful differences exist over time in terms of exercise adaptations and performance or recovery outcomes (*i.e.*, strength or time trial performance, glycogen resynthesis, muscle damage, and inflammation). Furthermore, when training or competition occurs late in the evening (e.g., many recreational sports and clubs) or early in the morning following an overnight fast (e.g., endurance events), the potential benefits of pre-sleep nutrition are evident.

### 3.2. Older Individuals

In today’s society people are living longer and the older population continues to grow at an alarming rate. In addition, older individuals have an increased risk of developing sarcopenia, the progressive loss of muscle mass and strength that increases the risk for physical disability, poor quality of life and reduced physical performance [62]. As aging and life expectancy are on the rise, multifactorial strategies aimed at attenuating sarcopenia to maintain functionality and quality of life are essential. Providing nutrients in close proximity to or during sleep to maintain and/or attenuate the loss of muscle mass may be a novel nutrition concept worthy of exploration.

To date, there is only one study that examined whether protein provided during sleep is properly digested and absorbed, and can subsequently improve overnight muscle protein synthesis in sixteen elderly men [45]. Due to the presence of a circadian variation in gastric emptying [2], it was speculated that providing nutrients at this time may not be efficiently utilized. Groen *et al.* administered 40 g of casein protein (intrinsically l-[1-13C]phenylalanine-labeled casein) through a nasogastric tube during sleep (0200–0205 h) and simultaneously assessed dietary protein digestion and absorption kinetics and muscle protein synthesis during overnight sleep *in vivo*. Consistent with findings in recreationally active men [52], this study reported that protein administration during sleep was well digested and absorbed and concomitantly promoted overnight muscle protein accretion in elderly men (Figure 1B) [45]. This find is interesting and highlights the potential significance of pre-sleep protein intake in populations susceptible to muscle loss and wasting (e.g., aging, cancer cachexia).

### 3.3. Obesity and Other Diseases

#### 3.3.1. Obesity

Given the growing interest in nighttime eating and its potential link to obesity and other cardiometabolic diseases, it is important to examine the impact of nighttime macronutrient choices on health outcomes in these populations. Waller *et al.* [46] demonstrated that providing a low fat, low calorie nighttime snack as opposed to the typical high fat/high calorie food option may be beneficial to overweight and obese adults. In this four week study, overweight and obese adults (*n* = 58; age, 18–65 years) were randomized to receive a structured, post-dinner snack consisting of cereal (1 cup of ready-to-eat cereal containing 100–135 kcals, 23–32 g carbohydrates, 2–6 g protein, <0.5g fat, and 1–1.5 g fiber) and 2/3 cup of low-fat milk (~60 kcals) or a control group that maintained their normal eating and post-dinner snacking behaviors (average snacking = 6.19 ±1.58 evenings per week, mean ± SD). The cereal was consumed every night at least 90 min after dinner for the duration of the study and the control group was offered the cereal after the initial four weeks. Findings from this study indicated that having a structured, post-dinner snack resulted in lower total daily caloric intake, evening caloric intake and modest weight loss (−0.84 ± 1.61 kg) in compliant individuals [46]. While not explicitly described, an interesting theory is that less food was consumed at dinner when it was known that an evening snack would be available.

In an acute study, Kinsey *et al.* [48] investigated the effect of different macronutrients (carbohydrate *vs.* protein) and proteins (casein *vs.* whey) consumed at night before sleep on next morning appetite and cardiometabolic risk in sedentary overweight and obese women. The single-macronutrient beverages (casein *vs.* whey *vs.* carbohydrate; 140–150 kcals) and supplementation protocol (two hours post-dinner but within 30 min of sleep) in this study were identical to those used by Madzima *et al.* [47]. This study revealed that these nighttime macronutrient beverages, regardless of type, lead to greater subjective satiety and less desire to eat. However, regardless of consumption of protein-only or carbohydrate-only, a small but significant increases in insulin levels and subsequent insulin resistance, as calculated using the homeostatic model of insulin resistance, was observed the morning following nighttime feeding [48]. The latter finding suggests that nighttime ingestion of protein or carbohydrate before sleep may elicit unfavorable metabolic effects in sedentary overweight and obese women. However, it was speculated that longer-duration interventions that combine pre-sleep nutrient intake with daily exercise may ameliorate this effect.

**Figure 3:**
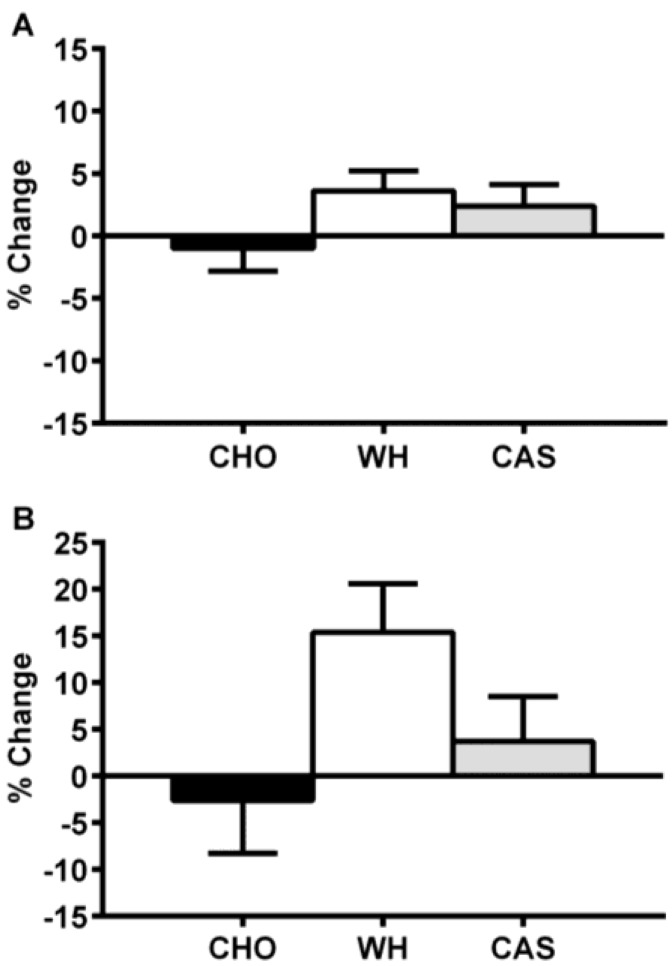
Resting Metabolic Rate (percent changes) Following Acute (**A**) and Four Weeks (**B**) of Nighttime Macronutrient Ingestion in Overweight and Obese Women. CHO, carbohydrate; WH, whey protein; CAS, casein protein. This figure was adapted and redrawn from Kinsey *et al.* [48] and Ormsbee *et al.* [49].

Interestingly, two recent studies [49,50] were designed to test the additive effect of four weeks of exercise training (3×/week; 2 days of resistance training and 1 day of high intensity cardiovascular interval training) and nighttime macronutrient ingestion (every night; beverages identical to those used by Madzima *et al.* [47] and Kinsey *et al.* [48]) on various health and performance outcomes. Ormsbee *et al.* [49] examined the additive effects of nighttime consumption of whey, casein or a carbohydrate beverage on appetite, cardiometabolic health and muscular strength in overweight and obese women (body mass index range: 25.7–47.5 kg/m^2^). As a result of exercise training, all groups showed modest, albeit significant, increases in muscle strength and lean mass, and decreased body fat (as measured by dual energy X-ray absorptiometry). Regarding appetite changes, greater morning satiety was reported in the group consuming casein at night compared to those consuming whey or carbohydrate. More importantly, the increase in morning insulin levels and insulin resistance observed acutely by Kinsey *et al.* [48] in response to nighttime protein and carbohydrate intake was abolished when exercise training was added to nighttime feeding in obese women [49]. Interestingly, although not statistically significant, it is noteworthy to mention that both acutely [48] and after four weeks [49] the direction of change for next morning metabolism was positive for the groups ingesting protein before sleep and negative for those ingesting carbohydrates (Figure 3). Considering that a higher protein diet has been shown to attenuate the typical drop in metabolism during sleep in overweight and obese adults, as compared to diets higher in carbohydrate and fat [63], these changes may have physiological relevance.

Moreover, Figueroa *et al.* [50] examined the impact of nighttime ingestion of whey or casein protein on blood pressure, and arterial function in young obese women with high blood pressure compared to a carbohydrate control group. This study demonstrated that the combination of nighttime protein supplementation and exercise training reduced both aortic systolic blood pressure, arterial stiffness (as measured by pulse wave velocity), and augmentation index compared to baseline and the carbohydrate beverage.

Overall, it appears that when exercise is combined with nighttime feeding of small, protein-rich nutrient intake before sleep, some positive physiological adaptations may occur in obese women. The benefit for other clinical populations, however, deserves some consideration.

#### 3.3.2. Glycogen Storage Disease and Diabetes

Patients with certain glycogen storage diseases (GSD) and those with Type 1 Diabetes (T1DM) are susceptible to nocturnal hypoglycemia and bedtime nutrient delivery provides an avenue for a supply of glucose during the night. GSDs are a group of genetic disorders that result from defects in enzymes required for glycogen synthesis or degradation [64]. Type I GSD is characterized by a deficiency in glucose-6 phosphatase activity, the liver enzyme required to dephosphorylate glucose to help maintain blood glucose levels. During the overnight period (*i.e.*, an extended fasting period) the liver is primarily responsible for maintaining blood glucose levels, however, the metabolic defect associated with Type 1 GSD impairs glycemic regulation. Accordingly, treatments for this condition are aimed at dietary regimens that provide a constant supply of glucose throughout the day and night to prevent hypoglycemia [64]. Studies have shown that individuals with Type I GSD can benefit from nighttime nutrient availability [53,65,66]. Traditional practice has been to provide uncooked cornstarch, a slow digesting carbohydrate and an effective source of continuous glucose for the prevention of hypoglycemia [67], at various intervals (2–5 h) throughout the day with nightly doses being administered by nasogastric feeding [66] or by interrupting sleep and waking the individual to eat [53]. The latter option is not ideal but, unfortunately, is most applicable and cost-effective in free-living adults with this condition. Thus, the efficacy of a pre-sleep option capable of sustaining blood glucose levels during sleep and hence prevent nocturnal hypoglycemia is immense. Wolfsdorf *et al.* [53] compared the effects of providing isoenergetic amounts of uncooked cornstarch orally on consecutive nights as single dose at bedtime or as two equally divided doses given at bedtime and mid-sleep in young adults with Type 1 GSD. The results demonstrated that blood glucose concentrations were maintained for 7 h and 9 h with the single and divided dose respectively. This highlights the feasibility of a bedtime dose of nutrients to aid in the maintenance of blood glucose concentrations during sleep in patients with Type 1 GSD.

T1DM is characterized by a defect in insulin production as a result of pancreatic beta cell destruction and, as a result, these individuals must rely on exogenous insulin in order to maintain appropriate blood glucose levels. Similar to those with Type 1 GSD, nocturnal hypoglycemia is also a concern for T1DM patients, particularly when bedtime glucose concentrations are ≤180 mg/dL (≤10 mmol/L) [54]. Likewise, bedtime feedings of cornstarch and/or protein have been useful in reducing hypoglycemic occurrences in populations with T1DM [54,68,69,70]. Kalergis *et al.* [54] examined the effect of bedtime ingestion of a standard snack (191 kcals, two starch and one protein exchange according the Academy of Nutrition and Dietetics), cornstarch snack (187 kcals), protein-rich snack (192 kcals), and no snack (0 kcals, aspartame-containing drink) on nocturnal hypoglycemia in patients with T1DM undergoing intensive insulin management. In this study, both the standard and protein-rich snacks were most successful at preventing nocturnal hypoglycemia while the no snack treatment had the greatest hypoglycemic episodes. However, although not significant, the incidence of next morning hyperglycemia in response to these nighttime snacks was greatest for the protein snack (33%), followed by the standard (29%) and cornstarch snack (29%), with the no snack treatment being the lowest (8%). It can be speculated that increased glucagon secretion and decreased insulin levels in the morning may have contributed the glycemia in this population [70]. Overall, this indicates that proper snack composition and individual variability may need to be considered when determining appropriate bedtime snacks for some clinical populations.

## 4. Conclusions

Old perspectives for nighttime eating have been primarily based on populations of shift workers, night eating syndrome patients, and epidemiological data and suggest that the consumption of large mixed meals combined with irregular sleep patterns increase susceptibility to weight gain, obesity, and cardiometabolic diseases. In recent years, data from healthy men has shown that consuming small ~150 kcal protein-rich beverages appears to improve overnight muscle protein synthesis, morning metabolism and satiety. However, the impact in healthy women has not been studied yet. In obese women, eating before sleep has been shown to improve morning appetite but also increase insulin resistance. However, the addition of exercise training for four weeks appears to eliminate any adverse effects of nighttime feeding in this population and has been shown to improve some indicators of cardiovascular health. In other diseased populations (e.g., GSD, T1DM), eating before bed is actually required for survival. However, management with individually tailored nighttime feeding protocols may optimize their clinical outcomes. Clearly, more research is required to examine this new perspective of nighttime feeding (small, nutrient dense, snacks before sleep) as it relates to sedentary individuals, athletes, and clinical populations. Moreover, identifying the role of nighttime feeding in recovery from exercise and improving performance, if any exist, are warranted along with identifying the overnight metabolic changes that may occur from nighttime feeding (*i.e.*, fat metabolism).
